# Targeting cancer cell-specific RNA interference by siRNA delivery using a complex carrier of affibody-displaying bio-nanocapsules and liposomes

**DOI:** 10.1186/1477-3155-11-19

**Published:** 2013-06-24

**Authors:** Yuya Nishimura, Hiroaki Mieda, Jun Ishii, Chiaki Ogino, Toshinobu Fujiwara, Akihiko Kondo

**Affiliations:** 1 Department of Chemical Science and Engineering, Graduate School of Engineering, Kobe University, 1-1 Rokkodai, Nada, Kobe 657-8501, Japan; 2 Organization of Advanced Science and Technology, Kobe University, 1-1 Rokkodai, Nada, Kobe 657-8501, Japan; 3 Laboratory of Hygienic Chemistry, Graduate School of Pharmaceutical Sciences, Nagoya City University, 3-1 Tanabe-dori, Mizuho-ku, Nagoya 467-8603, Japan

**Keywords:** RNA interference, siRNA, Hepatitis B virus, Bio-nanocapsule, Affibody, HER2

## Abstract

**Background:**

Small interfering RNA (siRNA) has attracted attention in the field of nucleic acid medicine as a RNA interference (RNAi) application that leads to gene silencing due to specific messenger RNA (mRNA) destruction. However, since siRNA is unstable in blood and unable to cross the cell membrane, encapsulation of siRNA into a carrier is required.

**Results:**

In this study, we used a carrier that combined Z_HER2_-displaying bio-nanocapsule (derived from hepatitis B virus surface antigen) and liposomes in a complex in order to investigate the feasibility of effective and target-cell-specific RNAi applications. As a result, by observing RNAi only in HER2-expressing breast cancer cells, using our proposed methodology, we successfully demonstrated target-cell-specific delivery and effective function expression of siRNA.

**Conclusions:**

These findings show that, in the field of nucleic acid medicine, Z_HER2_-BNC/LP can be a useful carrier for siRNA delivery, and could also become a useful tool for gene silencing and to accomplish protein knock-down.

## Background

RNA interference (RNAi) is expected to become a new approach in treating a variety of diseases, such as virus infection, cancer and neurodegenerative diseases, owing to specific and effective gene silencing [1,2]. The mechanism of RNAi involves double-stranded RNA injected into cells that are first cut into short RNA (small interfering RNA (siRNA)), 21–23 bp long, using ribonuclease (RNase) III enzyme that is referred to as the Dicer. The duplex siRNA forms a RNA-induced silencing complex (RISC), which contains an endonuclease and an Argonaute protein. The siRNA duplex is dissociated into unwound single-stranded RNA using an ATP-dependent helicase; therefore, the RISC with an antisense strand against target messenger RNA (mRNA) leads to RNA destruction and results in a downregulation of gene expression [3-6]. Although the use of siRNA is a promising approach for nucleic acid medicine, several problems remain with respect to its *in vivo* use, such as an inability to cross membranes, an instability in the blood, and a lack of ability to specifically target abnormal cells [1,7].

A bio-nanocapsule (BNC) is a hollow nano particle composed of the L protein of the hepatitis B virus (HBV), surface antigen (HBsAg), and a lipid bilayer. The BNC exhibits a reliable safety profile due to being viral-genome-free and shows high specificity for human hepatocytes and a high transfection efficiency that is equivalent to the original HBV [8]. Therefore, the BNC has been studied as a carrier for the delivery of drugs and genes [9].

Previously, we and other researchers succeeded in altering the cell-specificity of BNCs by deleting the hepatocyte-specific recognition site (located in the preS region) in the L protein and inserting binding molecules with the ability to target other cells [10]. For example, we have displayed the Z_HER2_ affibody molecule, which can specifically recognize human epidermal growth factor receptor 2 (HER2) expressed in breast and ovarian cells, as ligand on the surface of BNC (Z_HER2_-BNC). Affibody molecules (58 a.a.; 7 kDa) are new class of binding proteins derived from the Z domain of staphylococcal protein A and are consisted of three α-helices. Random mutation of 13 amino acid residues in N-terminal two helices can alter the recognition ability of affibody [11]. This led to successful alteration in the specificity of a BNC from hepatocytes to HER2 receptor expressing cells such as those found in breast and ovarian cancer [12].

Additionally, the fusion of medicinal proteins [13] and an electroporation [9] and a BNC/liposome (BNC/LP) conjugation [14] have been previously reported as methods used for encapsulating drugs and genes into BNCs. In particular, the BNC/LP conjugation method has succeeded in encapsulating various-sized materials including low-molecular compounds, genes and proteins into BNC/LP complex carriers [15,16]. The complex carriers are formed by fusing BNCs to the surface of LPs, in which target materials have been pre-encapsulated. By changing the phospholipid composition of LPs or the types of BNCs, a variety of characteristic features are granted to the BNC/LP complex carriers. For example, anionic phospholipid can avoid non-specific binding to non-target cells [16]; pH-responsive phospholipids (1,2-dioleoyl-sn-glycero-3-phosphoethanolamine; DOPE) provide the ability for endosomal escape [16]; and, affibody-displaying BNCs can alter cell-specificity [12]. By using this method, we previously constructed Z_HER2_-BNC/LP complex carriers and succeeded in the specific and functional delivery of proteins for HER2-expressing breast cancer cells [16].

**Figure 1:**
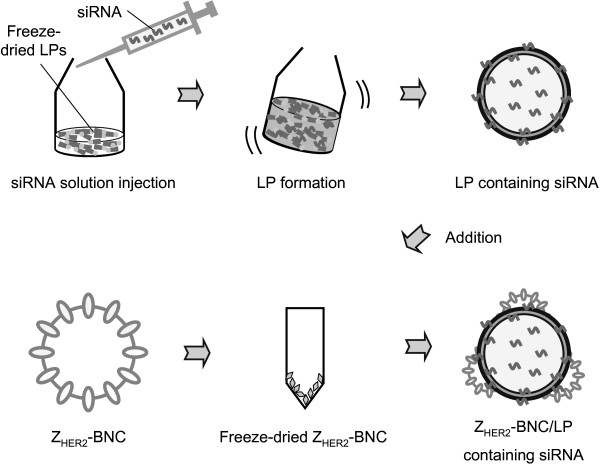
**Schematic illustration for conjugate formation of Z**_**HER2**_**-BNC/LP complex containing siRNA.**

In the present study, to overcome the problems in siRNA therapy, we tried the specific delivery of siRNA into target cancer cells by using the BNC/LP complex as the carrier (Figure 1). To facilitate the evaluation of RNAi, an siRNA that would inhibit GFP expression was selected. We describe how the Z_HER2_-BNC/LP complex can specifically deliver siRNA into HER2-expressing breast cancer cells and effectively lead to the cell-specific targeting of RNAi.

## Methods

### Materials

BNCs were prepared from *Saccharomyces cerevisiae* AH22R^-^ harboring either of the plasmids pGLDsLd50-Z_HER2_ or pGLDsLd50-Z_WT_[12], as described previously [8]. Briefly, yeast cells transformed with pGLDsLd50-Z_HER2_ or pGLDsLd50-Z_WT_ by the spheroplast method were cultured and disrupted with glass beads, the crude extract was precipitated with polyethylene glycol (PEG) 6000 and subjected to cesium chloride (CsCl) isopycnic ultracentrifugation and sucrose density gradient ultracentrifugation, and then the purified BNCs were obtained after freeze-drying in the presence of 5% sucrose. Liposome (LP), COATSOME EL-01-D (1,2-dioleoyl-*sn*-glycero-3-phosphoethanolamine (DOPE):CHOL:O,O’-ditetradecanoyl-N-(α-trimethylammonioacetyl) diethanolamine chloride (DC6-14) = 0.75:0.75:1.00 (μmol/vial)) was purchased from NOF (Tokyo, Japan). Silencer® GFP (eGFP) siRNA and Lipofectamin™ RNAiMAX Reagent was purchased from Invitrogen Life Technologies (Carlsbad, CA, USA). Amicon® Ultra-0.5 mL Centrifugal Filter Units (100,000 NMWL) were purchased from Merck Millipore (Massachusetts, USA). siRNA Ladder Marker was purchased from Takara Bio (Shiga, Japan). Gibco® Fetal bovine serum (FBS) and an l-glutamine and Molecular Probes® LIVE/DEAD® viability/cytotoxicity assay kit were purchased from Invitrogen Life Technologies. RPMI 1640 medium and Dulbecco’s modified Eagle medium (DMEM) were purchased from Nacalai Tesque (Kyoto, Japan). Blasticidin was purchased from InvivoGen (San Diego, CA, USA).

### Preparation of BNC/LP complex and incorporation of siRNA

Complex carriers of Z_HER2_-BNC and Z_WT_-BNC and LP, in which siRNA was incorporated, were prepared by referring to the previously described BNC/LP conjugation method with some modifications [14]. Freeze-dried LPs (COATSOME EL-01-D, 1.5 mg) were dissolved in distilled water (1 ml) containing 200 nM of siRNA. After incubation for 1 h at room temperature, LP-mixing siRNA (100 μl) was added to freeze-dried Z_HER2_-BNC or Z_WT_-BNC (50 μg as protein) and incubated at room temperature for 1 h to form BNC/LP complexes. The resultant complex carriers were named Z_HER2_-BNC/LP and Z_WT_-BNC/LP.

### Measuring the zeta potential and diameter of particles

The zeta potentials of LPs and a BNC/LP complex were determined using a Zetasizer Nano ZS (Malvern Instruments, Worcestershire, UK), following the manufacturer’s procedure.

### Evaluation of siRNA encapsulation efficiency

The siRNA solution (200 nM) and the mixture of Z_HER2_-BNC/LP and siRNA were quadruply concentrated using Amicon® Ultra-0.5 centrifugal filter units with a 100-kDa cutoff. Native (non-denaturing) polyacrylamide gel electrophoresis (native-PAGE) was performed (60 mA, Tris/glycine buffer) to determine free-siRNA, which could be never contained in the Z_HER2_-BNC/LP complex. After staining the gel with ethidium bromide, relative amounts of free-siRNA were determined using an ImageQuant LAS 4000 (GE Healthcare, Buckinghamshire, England), following the manufacturer’s procedure and by the following formula: V_lane3_/V_lane1_ × 100%. V_lane1_ and V_lane3_ were denoted as volume of band detected from quantitative imaging of the gel.

### Cell culture

To evaluate and quantify the RNAi efficacy, we used the cells that constitutively express GFP. SKBR3 cells (human breast carcinoma) were maintained in RPMI 1640 medium supplemented with 10% (v/v) FBS and 5 μg/ml Blasticidin at 37°C in 5% CO_2_. HeLa cells (human cervical carcinoma) were maintained in DMEM medium supplemented with 10% FBS and 5 μg/ml Blasticidin at 37°C in 5% CO_2_.

### Flow cytometric evaluation of RNAi and cell viability

Approximately 1 × 10^5^ SKBR3 and HeLa cells were seeded in 12-well plates and incubated 37°C for 24 h. After washing with serum-free medium, the required volumes of the complex carriers and LPs containing siRNA were added to the medium, and the volume was adjusted to 1 ml. Cells were incubated for 4 h, and then were washed twice with serum-free medium and cultured with FBS-containing medium for 44 h.

siRNA was also directly transfected to cells with RNAiMAX, following the manufacturer’s procedure. Briefly, the required volumes of 50 mM siRNA and 2 μl of RNAiMAX were added to 200 μl of serum-free medium in 12-well plates, and the medium was incubated for 20 min at room temperature. Then, 1 ml of serum-free medium containing cells (1 × 10^5^) was added to the 12-well plates. Cells were incubated for 4 h, washed twice with serum-free medium, and cultured with FBS-containing medium for 44 h.

To quantify RNAi efficacy, green fluorescence was detected and the decrement of GFP-expressing cells was counted. To quantify cell viability, red fluorescence was detected and the dead cells stained with EthD-1 were counted. The EthD-1 staining was performed using a LIVE/DEAD® viability/cytotoxicity assay kit according to the manufacturer’s instructions. Cells were suspended into a sheath solution and subjected to a BD FACSCanto II flow cytometer equipped with a 488-nm blue laser (BD Biosciences, San Jose, CA, USA). The green and red fluorescence signals were collected through 530/30 and 585/42-nm band-pass filters, respectively. The data were analyzed using BD FACSDiva software v5.0 (BD Biosciences).

### Microscopic observation of GFP-expressing cells treated with siRNA

The introduction of siRNA basically followed the above-described procedure with some modifications: 12-well plates were changed to 35 mm glass bottom dishes; the final concentration of siRNA in the medium was fixed to 25 nM; and, the total volume of medium was adjusted to 2 ml.

Cells were observed using a LSM 5 PASCAL laser scanning confocal microscope (Carl Zeiss, Oberkochen, Germany) equipped with a 63-fold oil immersion objective lens with excitation using the 488-nm line of an argon laser and emission collection using a 505-nm long pass filter.

## Results

### Target cell-specific RNAi with Z_HER2_-BNC/LP

**Figure 2:**
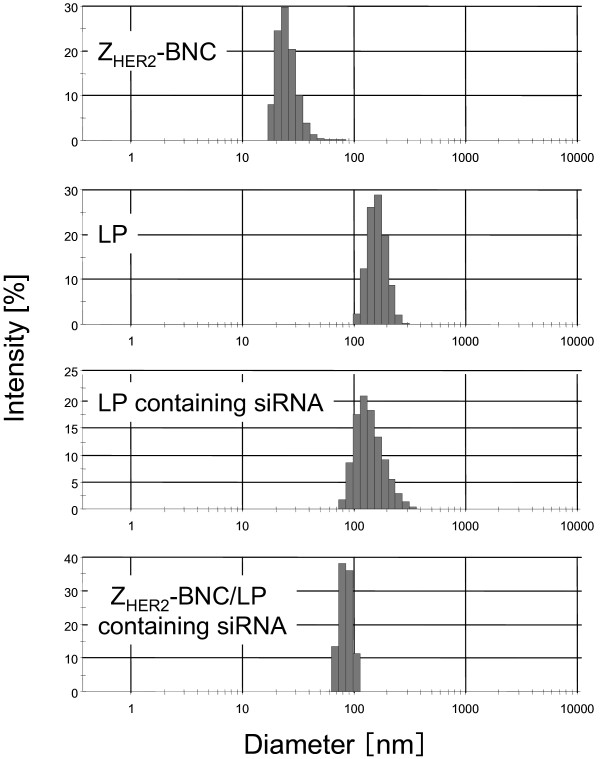
**The DLS analyses of Z**_**HER2**_**-BNC, LP, LP containing siRNA and Z**_**HER2**_**-BNC/LP containing siRNA.**

First, to examine the specific delivery of siRNA, we used HER2-expresing SKBR3 cells (human breast carcinoma) as target cells [17]. HER2-non-expressing HeLa cells (human cervical carcinoma) were used as the non-target cells [18]. To evaluate and quantify the RNAi efficacy, we used the cells constantly expressing the chromosomally-integrated GFP. RNAiMAX, LPs and Z_HER2_-BNC/LP were tested to deliver silencer GFP siRNA which can specifically interfere with the expression of GFP. The diameter, zeta potential and particle size distribution of each particle are shown in Table 1 and Figure 2. Since the different particle size distributions between Z_HER2_-BNC and LP containing siRNA were integrated into indistinguishable single distribution, it seemed that nearly every Z_HER2_-BNC and LP was conjugated (Figure 2). Because Z_HER2_-BNC has membrane fusion domains derived from L protein [14], it would appear that the lipid-lipid conjugation between Z_HER2_-BNC and LP has occurred efficiently. The particle size distribution of Z_HER2_-BNC/LP remained the same after incubation for 10 days at 4°C (data not shown).

**Table 1:** **The sizes and zeta potentials of Z**_**HER2**_**-BNCs only, LPs only, LPs containing siRNA and Z**_**HER2**_**-BNC/LP complex containing siRNA**

**Carrier**	**Diameter [nm]**	**Zeta potential [mV]**
Z_HER2_-BNC	25 ± 1.3	−23.7 ± 2.4
LP	155 ± 11.3	3.9 ± 1.8
LP containing siRNA	134 ± 13.0	1.4 ± 2.9
Z_HER2_-BNC/LP containing siRNA	97 ± 18.0	−5.1 ± 1.2

**Figure 3:**
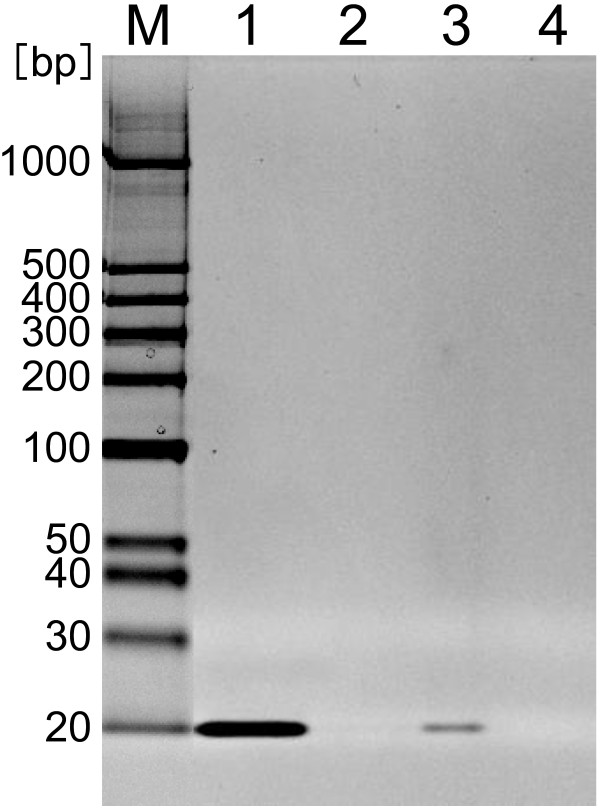
**Analysis of siRNA encapsulation efficiency by native-PAGE.** Lane 1 and lane 2 show quadruple concentrated solution of siRNA (200 nM) and its waste solution, respectively. Lane 3 and lane 4 show quadruple concentrated mixture of Z_HER2_-BNC/LP containing siRNA and its waste solution, respectively.

Second, to evaluate encapsulation efficiency of siRNA into Z_HER2_-BNC/LP, we analyzed free-siRNA by native-PAGE (Figure 3). Since the siRNA contained in Z_HER2_-BNC/LP particle never migrated into the gel, the free-siRNA from Z_HER2_-BNC/LP should appear as a 20-mer band after electrophoresis. By comparing the degree of reduction between the band in lane 1 (siRNA solution without LP) and that in lane 3 (free-siRNA from Z_HER2_-BNC/LP), the encapsulation efficiency of siRNA into Z_HER2_-BNC/LP could be estimated. As the result of measurement for the degree of siRNA reduction using ImageQuant LAS 4000, encapsulation efficiency of siRNA was 94.4%. This demonstrated that it was possible to encapsulate siRNA efficiently into Z_HER2_-BNC/LP.

**Figure 4:**
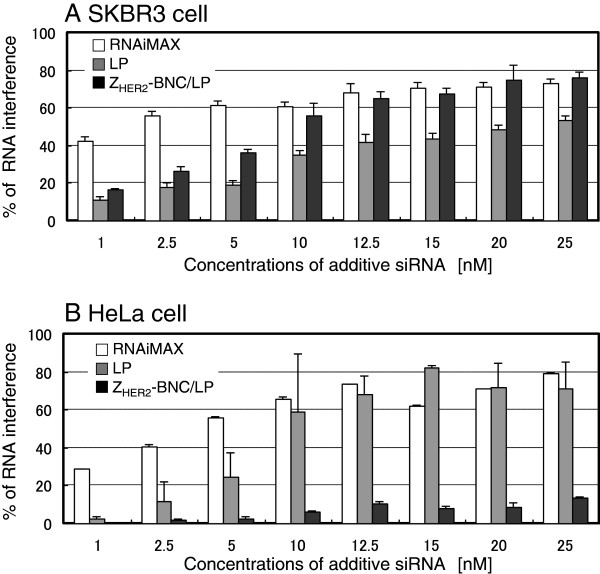
**Quantification of RNAi in HER2-positive SKBR3 (A) and HER2-negative HeLa (B) cells treated by siRNA combined with RNAiMAX (white bars), LPs (gray bars) and Z**_**HER2**_**-BNC/LP complex (black bars).** The GFP expressions of the cells were analyzed using a flow cytometer and results are expressed as a percentage of the GFP-expressing cellular quantity in untreated controls. The x-axis represents the final concentration of siRNA in the medium adjusted to 2 ml.

After 48 h of incubation, the efficacies of RNAi depending on the additive concentration of siRNA were determined by measuring the cell population rates missing GFP fluorescence. The efficacies of RNAi for HER2-expressing SKBR3 cells and HER2-non-expressing HeLa cells are shown in Figures 4A and 4B, respectively. In the case of using RNAiMAX (white bars), RNAi was observed even at lower concentrations of siRNA (1 nM~) in both SKBR3 and HeLa cells. This indicated that the transfection reagent never showed specificity to the target cells, although it has the ability for high transfection efficiency. The LPs without Z_HER2_-BNC (gray bars) also triggered RNAi in both cells, similar to the case of RNAiMAX. The zeta potential of LPs encapsulating siRNA showed a positive charge (Table 1), suggesting that it was bound to cells non-specifically due to an electrostatic interaction. However, the Z_HER2_-BNC/LP complex displayed the specific effect of RNAi only for HER2-expressing SKBR3 cells (Figures 4A and 4B; black bars). Furthermore, the RNAi efficacies of Z_HER2_-BNC/LP that were >10 nM were equal to that of RNAiMAX. This result indicates that the siRNA delivery with Z_HER2_-BNC/LP was HER2-expressing breast cancer cell-specific siRNA delivery, and that it led to an effective expression of the RNAi function.

### Viability of cells treated with Z_HER2_-BNC/LP

**Figure 5:**
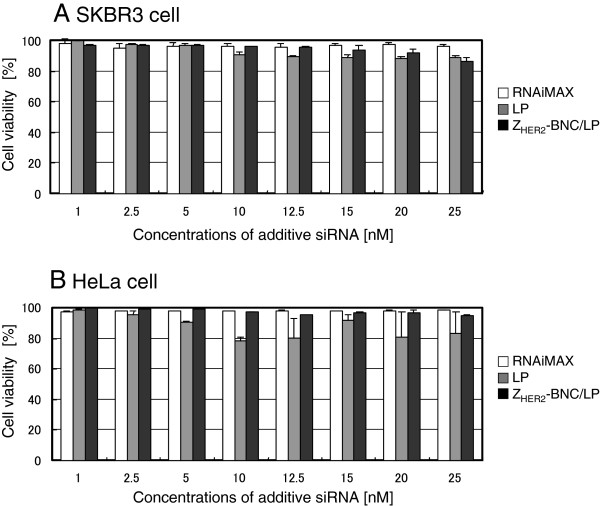
**Cell survival rates of HER2-positive SKBR3 (A) and HER2-negative HeLa (B) cells treated by siRNA combined with RNAiMAX (white bars), LPs (gray bars) and Z**_**HER2**_**-BNC/LP complex (black bars).** The fluorescence of cells stained with EthD-1 was analyzed using a flow cytometer. Cell survival rates were calculated by subtracting the dead cell counts from the total cell counts.

To evaluate the biocompatibility of each carrier containing siRNA, we measured cell survival rates with a type of EthD-1 that permits the detection of dead cells under the progression of RNAi. The cell viabilities of SKBR3 (Figure 5A) and HeLa (Figure 5B) were similar, but slightly different for each carrier. The slight decrease in the viability of LPs (gray bars) might have been due to the excess non-specific binding of phospholipids to the cell membrane. A significant decrease in viability was not observed for Z_HER2_-BNC/LP (black bars). This result suggests that Z_HER2_-BNC/LP was non-toxic to cells.

### Confirmation of the occurrence of siRNA-specific and affibody-dependent RNAi

**Figure 6:**
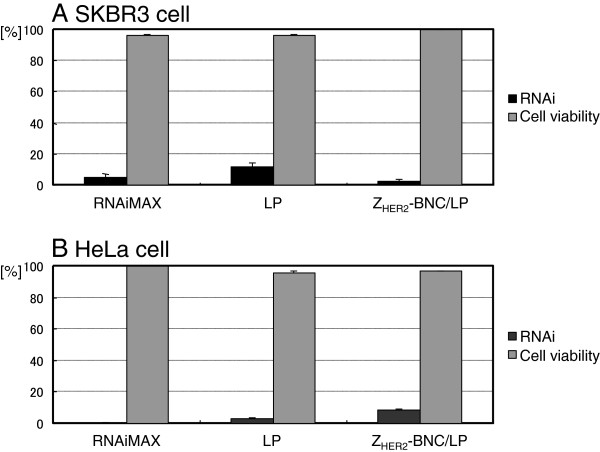
**Quantification of RNAi (black bars) and cell survival rates (gray bars) of HER2-positive SKBR3 (A) and HER2-negative HeLa (B) cells treated by negative siRNA combined with RNAiMAX, LPs and Z**_**HER2**_**-BNC/LP complex (final conc. 25 nM as siRNA).**

To confirm whether the inhibition of protein synthesis was really led by the action of siRNA, we used the siRNA that never inhibits GFP expression (negative siRNA) (Figure 6). We added 25 nM carriers containing negative siRNA to SKBR3 and HeLa cells. As a result, RNAi was never detected in the presence of any of these carriers (Figures 6A and 6B). This result clearly shows that the decrement of GFP-expressing cells in Figure 4 was surely guided by siRNA-specific action.

**Figure 7:**
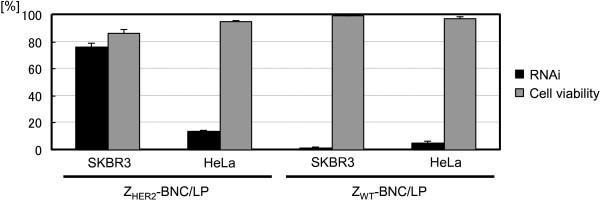
**Quantification of RNAi (black bars) and cell survival rates (gray bars) of HER2-positive SKBR3 and HER2-negative HeLa cells treated by siRNA combined with Z**_**HER2**_**-BNC/LP (left side) and Z**_**WT**_**-BNC/LP (right side) (final conc. 25 nM as siRNA).**

To establish the validity of using Z_HER2_-BNC to grant cell-specificity, we compared Z_HER2_-BNC/LP with Z_WT_-BNC/LP using the Z_WT_ (Z domain)-displaying BNC without HER2 recognition ability to form the BNC/LP complex (Figure 7). Each complex carrier with siRNA was added to SKBR3 and HeLa cells (final conc. 25 nM as siRNA), and the rates of RNAi and cell viability were evaluated after 48 h of incubation. As a result, Z_HER2_-BNC/LP triggered SKBR3-specific RNAi, whereas Z_WT_-BNC/LP did not invoke RNAi in either cell. Thus, the importance of the affibody-displaying BNC for specific siRNA delivery was confirmed.

### Microscopic observation of GFP interference

**Figure 8:**
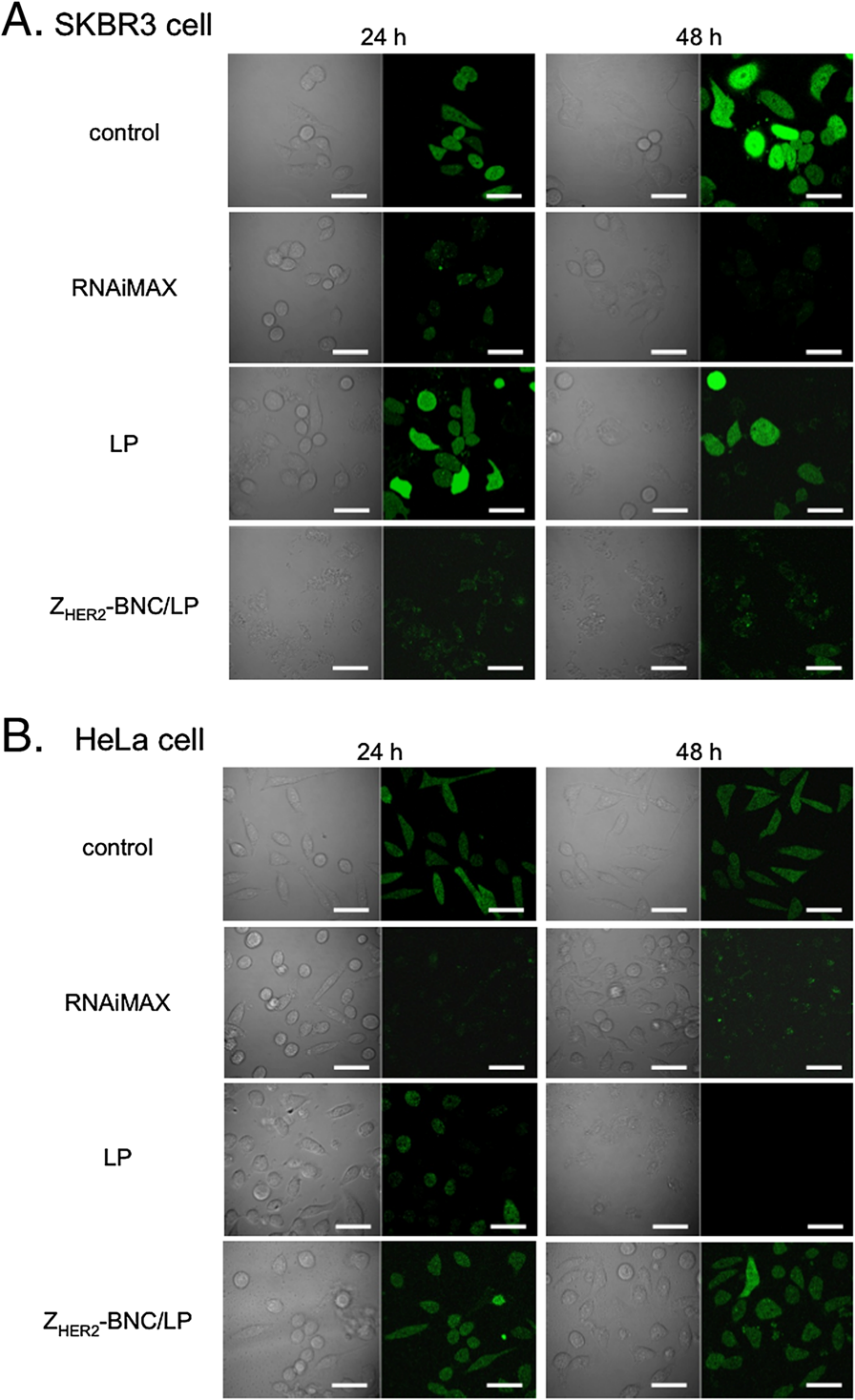
**Fluorescence images of HER2-positive SKBR3 (A) and HER2-negative HeLa (B) cells treated by siRNA combined with RNAiMAX, LPs and Z**_**HER2**_**-BNC/LP complex (final conc. 25 nM as siRNA).** The cells were incubated for 24 and 48 h, and then observed using a confocal laser scanning microscope. Scale bars, 50 μm.

To visually confirm the inhibition of GFP synthesis by RNAi, we treated SKBR3 and HeLa cells with RNAiMAX, LPs and Z_HER2_-BNC/LP containing siRNA (final conc. 25 nM) and observed the cells using a confocal laser scanning microscope (CLSM) following 24 and 48 h of incubation (Figures 8A and 8B). In the case of RNAiMAX, green fluorescence was rarely observed in either cell, and the non-specific inhibition of GFP synthesis was confirmed. In the case of LP, inhibition of GFP synthesis was scarcely provoked after 24 h but was confirmed after 48 h in both cells. This indicated that LPs would bind to cells in a non-specific manner, and it took longer to induce RNAi than with the transfection reagent. However, Z_HER2_-BNC/LP had no impact on the expression of GFP in HeLa cells, while the inhibition of GFP synthesis was clearly confirmed in SKBR3 cells during 48 h of incubation. Furthermore, diminished GFP fluorescence was observed even after 24 h, indicating that Z_HER2_-BNC/LP had fast-acting properties that were equivalent to the transfection reagent. These results demonstrated that Z_HER2_-BNC/LP can stabilize siRNA via the formulation of a complex carrier to efficiently deliver siRNA inside specific HER2-expressing cells through endosomal escape, which would allow RNAi to effectively inhibit protein expression.

## Discussion

Presently, various carriers delivering siRNA have been studied and many research papers have reported using the inhibition of GFP synthesis as a measure of RNAi. In the present study, we succeeded in specifically delivering siRNA to SKBR3 cells using a complex carrier of BNCs and LPs (Z_HER2_-BNC/LP), and measured an 80% inhibition of GFP synthesis following a 48 h period of incubation (Figure 4A; siRNA 20 nM).

In previous studies, PEI-PEG-FOL using cationic polymers, cationic dextran nanogel using nanogel and AF-AuNPs/siRNA-PEG using Au nanoparticles inhibited 80% of GFP synthesis after 48 h of incubation with a siRNA of 0.5 μg/ml [19], after 72 h of incubation with a siRNA of 50 nM [20], and after 24 h of incubation with a siRNA of 60 nM [21], respectively. Furthermore, in the case of lipoplex using cationic phospholipids, LinOS/Chol and LinOS/DOPE inhibited 80% of GFP synthesis after 48 h of incubation with a siRNA of 15 nM [22]. The results of these studies suggest that Z_HER2_-BNC/LP is effective at relatively low concentrations of siRNA. Additionally, since the results of CLSM showed equal efficacy between incubations of 24 and 48 h (Figure 8A), it could be concluded that Z_HER2_-BNC/LP shows a relatively early effective time for the inhibition of GFP synthesis.

Compared to the carriers mentioned above, the greatest advantage of Z_HER2_-BNC/LP is cell-specificity to target cells. Since Z_HER2_-BNC/LP displays Z_HER2_ on the surface, it was confirmed that Z_HER2_-BNC/LP delivers siRNA to HER2-expressing breast cancer cells (SKBR3), but not to HER2-non-expressing cervical cancer cells (HeLa) (Figures 4 and 8). Since BNC/LP complexes can recognize receptors other than HER2 by altering ligands displayed on the BNCs to other affibodies or peptides, it should be possible to alter the specificity, the conjugation of BNCs with LPs plays an important role for the stability arising from stealth capability of virus vector in the blood and blocking non-specific electrostatic interaction with the cell membrane.

Since siRNA has a negative charge itself, the positively charged materials (polymer, sugar chain and lipid) have been commonly used for forming complexes. Therefore, carriers often show a positive charge in total. In zeta potential, for example, since PEI-PEG-FOL, AF-AuNPs/siRNA-PEG and LinOS/Chol and LinOS/DOPE showed 2.5 ± 0.9 mV [19], 35.7 ± 8.1 mV [21], and approximately 60 mV [22], respectively, the carriers were usually introduced into cells by using electrostatic interaction with a cell membrane. The zeta potential of Z_HER2_-BNC/LP, on the other hand, showed a negative charge, -5.1 ± 1.2 mV (Table 1). Therefore, from the point of view of suppression of non-specific adsorption to a cell membrane, Z_HER2_-BNC/LP could be specifically introduced into HER2-expressing cells by a cell recognition site derived from BNC.

Furthermore, cationic carriers are often modified by polyethylene glycol (PEG) to improve stability in the blood. However, since there is the dilemma that the stability obtained by modifying the PEG that forms hydration layers would cause a decrease in cellular dynamics such as transfection efficiency and endosomal escape, these carriers require well-balanced control between disposition and cellular dynamics [23]. With Z_HER2_-BNC/LP it is not a problem, however, since there is no required modification of PEG due to a carrier having a negative charge. Indeed, since the BNC/LP complex carrier is made stable by the virus envelope protein and evaluated *in vivo* using mice without modification of PEG [15], Z_HER2_-BNC/LP is expected to have a stability that is similar to BNC/LP. Since the disposition plays a significant factor in drug delivery systems, we thought that Z_HER2_-BNC/LP would show promising results during the clinical application of siRNA delivery.

One important issue for the Z_HER2_-BNC/LP is that the efficiency of RNAi has not yet reached the same level of the RNAiMAX transfection reagent found in low concentrations of siRNA (Figure 4A). This is because the efficiency of endosomal escape remains low as long as the siRNA remains in the endosome. To improve the ability of endosomal escape to Z_HER2_-BNC/LP, we mixed pH-response phospholipids (DOPE) with LP. However, by further enhancing the ability of endosomal escape, Z_HER2_-BNC/LP would be expected to lead RNAi in low concentrations, as is the case with transfection reagents.

## Conclusions

Although the therapeutic effect of siRNA has been highly anticipated, its inability to specifically target cells and to cross the cell membrane has limited its *in vivo* application [1]. In this study, we succeeded in delivering and introducing siRNA into targeted breast carcinoma cells, which led to the effective use of RNAi by using Z_HER2_-BNC/LP as the carrier. Thus, in the field of nucleic acid medicine, Z_HER2_-BNC/LP can be a useful carrier for siRNA delivery, and could also become a useful tool for gene silencing and to accomplish protein knock-down.

## Abbreviations

RNAi: RNA interference; siRNA: Small interfering RNA; RNase: Ribonuclease; RISC: RNA-induced silencing complex; BNC: Bio-nanocapsule; HBV: Hepatitis B virus; HBsAg: Hepatitis B virus surface antigen; LP: Liposome; DOPE: 1,2-dioleoyl-sn-glycero-3-phosphoethanolamine; GFP: Green fluorescent protein; HER2: Human epidermal growth factor receptor 2; WT: Wild type, PAGE: poly-acrylamide gel electrophoresis; CLSM: Confocal laser scanning microscope; PEI-PEG-FOL: Poly(ethylenimine)-*graft*-poly(ethylene glycol)-folate; AF-AuNPs: Amine-functionalized gold nanoparticles; LinOS/Chol: N^4^-linoleoyl-N^9^-oleoyl-1,12-diamino-4,9-diazadodecane/cholesterol; CsCl: Cesium chloride; FBS: Fetal bovine serum; DMEM: Dulbecco’s modified Eagle medium; EthD-1: Ethidium homodimer-1.

## Competing interests

The authors declare that they have no competing interests.

## Authors’ contributions

Conceived and designed the experiments: YN, HM, JI, CO and TF. Performed the experiments: YN and HM. Analyzed the data: YN and HM. Wrote the paper: YN and JI. Supervised the whole work: AK. All authors read and approved the final manuscript.
